# Integrative analyses of bulk and single-cell transcriptomics reveals the infiltration and crosstalk of cancer-associated fibroblasts as a novel predictor for prognosis and microenvironment remodeling in intrahepatic cholangiocarcinoma

**DOI:** 10.1186/s12967-024-05238-z

**Published:** 2024-05-03

**Authors:** Yan-Jie Zhong, Xi-Mei Luo, Fei Liu, Zhi-Qiang He, Si-Qi Yang, Wen-Jie Ma, Jun-Ke Wang, Yu-Shi Dai, Rui-Qi Zou, Ya-Fei Hu, Tian-Run Lv, Fu-Yu Li, Hai-Jie Hu

**Affiliations:** 1 Division of Biliary Tract Surgery, Department of General Surgery, West China Hospital, Sichuan University, Chengdu, 610041 Sichuan Province China; 2 Institute of Fundamental and Frontier Sciences, University of Electronic Science and Technology of China, Chengdu, Sichuan China

**Keywords:** WGCNA, Single cell transcriptomic, Intrahepatic cholangiocarcinoma, Cancer-associated fibroblasts, Intercellular communication, Prognosis

## Abstract

**Background:**

Intrahepatic cholangiocarcinoma (ICC) is a highly malignant neoplasm and characterized by desmoplastic matrix. The heterogeneity and crosstalk of tumor microenvironment remain incompletely understood.

**Methods:**

To address this gap, we performed Weighted Gene Co-expression Network Analysis (WGCNA) to identify and construct a cancer associated fibroblasts (CAFs) infiltration biomarker. We also depicted the intercellular communication network and important receptor-ligand complexes using the single-cell transcriptomics analysis of tumor and Adjacent normal tissue.

**Results:**

Through the intersection of TCGA DEGs and WGCNA module genes, 784 differential genes related to CAFs infiltration were obtained. After a series of regression analyses, the CAFs score was generated by integrating the expressions of EVA1A, APBA2, LRRTM4, GOLGA8M, BPIFB2, and their corresponding coefficients. In the TCGA-CHOL, GSE89748, and 107,943 cohorts, the high CAFs score group showed unfavorable survival prognosis (*p* < 0.001, *p* = 0.0074, *p* = 0.028, respectively). Additionally, a series of drugs have been predicted to be more sensitive to the high-risk group (*p* < 0.05). Subsequent to dimension reduction and clustering, thirteen clusters were identified to construct the single-cell atlas. Cell-cell interaction analysis unveiled significant enhancement of signal transduction in tumor tissues, particularly from fibroblasts to malignant cells via diverse pathways. Moreover, SCENIC analysis indicated that HOXA5, WT1, and LHX2 are fibroblast specific motifs.

**Conclusions:**

This study reveals the key role of fibroblasts - oncocytes interaction in the remodeling of the immunosuppressive microenvironment in intrahepatic cholangiocarcinoma. Subsequently, it may trigger cascade activation of downstream signaling pathways such as PI3K-AKT and Notch in tumor, thus initiating tumorigenesis. Targeted drugs aimed at disrupting fibroblasts-tumor cell interaction, along with associated enrichment pathways, show potential in mitigating the immunosuppressive microenvironment that facilitates tumor progression.

## Introduction

Intrahepatic cholangiocarcinoma originates from the epithelial cells of the intrahepatic bile ducts and holds the second position in incidence after hepatocellular carcinoma, comprising 10–15% of primary liver cancer [1]. Reports indicate that China contributes to nearly half of the global annual incidence and mortality rates of liver cancer. Given that the risk factors for intrahepatic cholangiocarcinoma remain incompletely understood, it may continue to pose a significant threat to the majority of patients [2, 3]. Thereinto, mass-forming ICC is notably distinguished by a substantial desmoplastic component composed of fibroblasts and a hypovascularized tumor stroma [4].

Surgical resection stands as the sole potentially curative treatment, but approximately 20-30% of patients are resectable and relapse rate remains high [5]. In addition to this, chemotherapy remains the most prevalent non-surgical treatment modality for various types of cancers worldwide [6]. For most ICC patients with unresectable status or distant metastases at diagnosis, the cornerstone of treatment involves chemotherapy with cisplatin and gemcitabine, followed by second-line options like 5-FU and oxaliplatin/irinotecan [7]. However, tumor heterogeneity is the main reason for the modest efficacy of chemotherapy, resulting in an objective response rate of merely 5% and a median overall survival of 6.2 months [8, 9]. Unfortunately, the inevitable side effects should not be ignored [10]. Combine systemic therapy with locoregional therapies, and even molecular therapy is gradually developing [11]. Increasing number of researches are being dedicated to the synthesis of novel compounds or the extraction of active components from natural products, aiming to provide new avenues for non-surgical tumor treatment [12–15].

Additionally, CCA is characterized by intense desmoplastic stroma, in which the dominant cellular population is tumor-associated fibroblasts [16]. The presence of a dense collagenous stroma serves as a robust indicator of tumor aggressiveness and therapeutic resistance [17]. In the liver, CAFs are derived from portal fibroblasts or hepatic stellate cells (HSCs), and activated CAFs are associated with larger tumor size and poor prognosis [4]. Enhanced secretion of matrix proteins, pro-invasive factors, cytokines, and matrix-modifying enzymes by CAFs can intensify malignancy and therapeutic resistance in cholangiocarcinoma [4]. Evidence suggests that significantly lower 1- and 3-year survival rates in tumors with abundant fibrous stroma compared to ICC with a sparse fibrous matrix [18]. Moreover, nab-paclitaxel was proven to be an effective treatment for desmoid-like intrahepatic cholangiocarcinoma by destroying cancer-associated fibroblasts [19]. Considering the stroma mechanical properties of ICC, gold nanoparticles (GIONF)-mediated photothermal therapy was reported to deplete CAFs and normalize the tumor mechanics [20]. Therefore, we speculate that targeting fibroblasts and their secretions, as well as the desmoplastic matrix could be a potential alternative therapy option.

Increasing evidence showed the heterogeneity of the tumor microenvironment and its important role in promoting tumor occurrence and treatment resistance [21]. In addition, Single-cell transcriptomics reveals intricate intercellular communication networks within diverse cell types in the TME [22, 23]. Nevertheless, there remains limited exploration of the combined impact of intercellular communication and signaling pathway activation in microenvironment remodeling and tumor development. In our study, we noted a pronounced link between fibroblasts and poorer prognosis in ICC compared to other immune cells. The fibroblast infiltration-related genes obtained by WGCNA analysis were further narrowed down to establish a prognostic model. Subsequently, single-cell transcriptomics unveiled notable distinctions in intercellular transduction between the tumor and normal groups, and found the signal transmission from fibroblasts to malignant cells obviously enhanced in tumor tissues. Differentially over-expressed ligands and receptors-mediated signal transmission also were identified.

## Materials and methods

### Data collection

To construct the risk model, we extracted TCGA-CHOL samples with complete clinical information from the UCSC Xena database (https://xena.ucsc.edu) as the model training set. For external validation, ICC samples from the GEO database (GSE89748 and GSE107943) were employed. Additionally, scRNA-seq data for ICC patients were sourced from GEO (https://www.ncbi.nlm.nih.gov/geo/, accession number GSE138709).

The construction of the microenvironmental cell atlas began with marker gene identification and cell classification through Seurat. The dataset consisted of 8 samples from 5 ICC patients, including 5 tumor samples and 3 adjacent normal tissues.

### Calculation of CAFs scores

Quantification of immune cell infiltration abundance in the immune microenvironment was carried out using the MCP-counter algorithm implemented in the IOBR package. Then, we categorized samples from the TCGA cohort into two subgroups based on the median CAFs score. The heatmap between immune cell infiltration score and clinical features was established to determine the immune cell subtypes with the strongest correlation with clinical prognostic indicators.

### Differentially expressed gene (DEG) analysis and weighted gene coexpression network analysis (WGCNA) for module identification

Application of the R package “limma” enabled Differential Gene Expression (DEG) analysis, where genes with a *p*-value < 0.05 and |Log2 (fold change (FC))| > 1 were designated as DEGs. Additionally, we utilized Weighted Gene Coexpression Network Analysis (WGCNA) to pinpoint coexpressed gene modules most significantly linked to CAF infiltration. The hub genes emerged through the intersection of DEGs and CAFs-related gene modules.

### Generation and validation of CAFs-related prognostic biomarkers

Applying Univariate Cox analysis, we identified genes linked to overall survival (OS) in TCGA-CHOL patients, specifically focusing on hub genes. To further narrow down candidates, we employed LASSO and multivariate Cox regression to select significant predictors. This allowed us to generate polygenic risk scores, enabling the division of TCGA-CHOL samples into low- or high-risk subgroups. The formula for score calculation was as follows:

risk score = (coefficient of Gene 1 × expression of Gene 1) + (coefficient of Gene 2 × expression of Gene 2) +···+ (coefficient of Gene n × expression Gene n).

Our evaluation of predictive performance involved the generation of time-dependent receiver operating characteristic (ROC) curves. The analysis and visualization were conducted using R packages, including survival, survminer, rms, and time ROC.

### Drug prediction

We utilized the ridge regression model from the ‘pRRophetic’ package to predict drug response. This allowed us to conduct a comparison of drug sensitivity between groups with high and low CAFs scores.

### scRNA-seq data processing

Analysis of scRNA-seq data was conducted using the Seurat package in R software (version 4.1.2 and 4.3.1). Samples with over 10% mitochondrial genes and 3% ribosomal genes were excluded. We also filtered out the genes expressed in fewer than three cells, and cells with fewer than 200 or more than 6,000 expressed genes. The FindVariableFeatures function in Seurat identified the most variable genes. Principal Component Analysis (PCA) was utilized for dimensionality reduction. To remove the batch effects, the harmony R package was employed. A total of 13 clusters were identified by tSNE cluster analysis, and cell types were finally annotated by combining reported and most changed marker genes.

### Analysis of intercellular communications

We employed the CellChat package to explore potential disparities in intercellular interactions between tumor and normal samples following the official workflow. It could mimics intercellular communication by evaluating binding ligands, receptors, and their respective cofactors. Inferences regarding interactions between the two cell types were based on receptor expression in one type and ligand expression in the other. The visualization of signaling pathways was achieved through the ‘netVisual_aggregate’ function. Ligands and receptors were respectively defined as efferent and afferent signals.

### Functional enrichment analysis

KEGG or GO analysis of gene sets of interest to understand functional differences performed by different groups and cell types. Moreover, we additionally utilized the ‘scMetabolism’ package to compare the metabolic difference across various cell types. The metabolic activity of each cell was quantified through integrating the gene sets contained in the KEGG database.

### The analysis of transcription factor regulatory network

PySCENIC was employed for the analysis of the regulatory network and regulon activity. A regulon, comprising a transcription factor and its direct target genes, was established. The AUCell module within pySCENIC facilitated the analysis of regulon activity, with active regulons identified using the default AUCell threshold. Additionally, a heatmap was generated using the scaled expression of regulon activity. Further insights into regulon specificity for each cell type were obtained through regulon-specific scores (RSS).

## Results

### WGCNA of gene sets associated with tumor-associated fibroblast infiltration

**Fig. 1:**
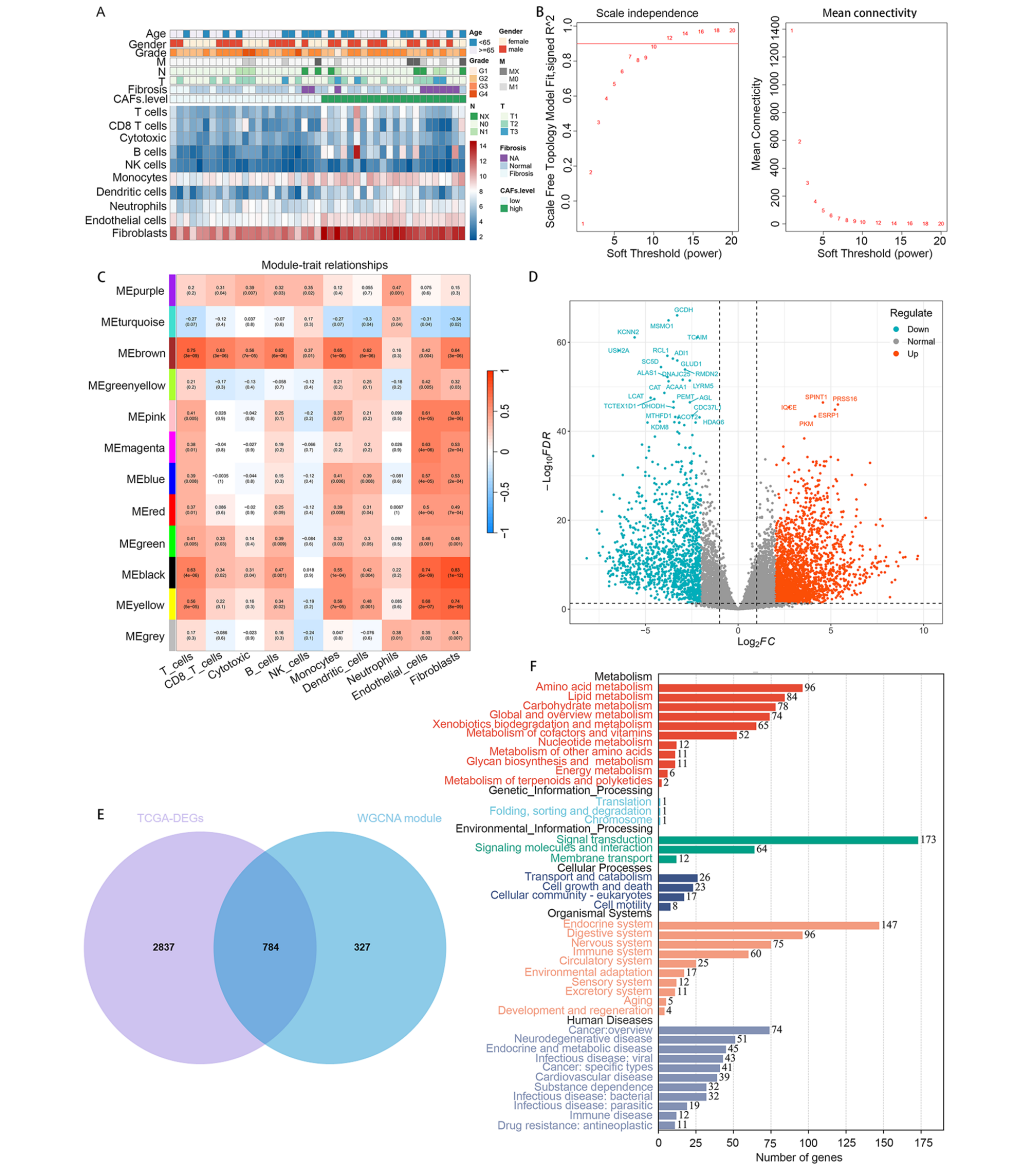
Screening of CAFs-inflitration related genes. (**A**) Various TME cell abundances in the TCGA cohort are shown in the heat map. Associations between CAFs level and clinicopathological characteristics are also illustrated as an annotation. (**B**) The nature of the network topology constructed with unique power values. (**C**) The correlation between different modules and the proportion of CAFs-high and low infiltration. (**D**) Volcano plot of differentially expressed genes (DEGs) in TCGA-CHOL. (**E**) Venn plot shows the hub genes intersected by DEG and WGCNA. (**F**) KEGG functional enrichment analysis of hub genes in TCGA-CHOL dataset

To explore the proportion of immune cells in cholangiocarcinoma and their role in tumorigenesis and development, we initially employed MCP-counter algorithm to estimate the abundance of fibroblasts in the TCGA-CHOL dataset. We subsequently found that fibroblasts showed a more significant association with clinical adverse outcomes than other immune cells. In addition, the abundance of CAFs exhibited a notable increase in patients at advanced TNM stage (Fig. 1A). We conducted weighted gene co-expression analysis (WGCNA) on cholangiocarcinoma (CHOL) samples from the TCGA database for identifying gene module associated with CAFs infiltration, which involved the construction of co-expression networks and the identification of co-expression modules (Fig. 1B). Pearson tests were employed to assess correlations between CAF infiltration scores and modules. Notably, the black and turquoise modules (1111 genes in total) demonstrated the highest strongest correlation with the clinical indications and CAFs infiltration (Fig. 1C). These two modular gene sets were intersected with the TCGA differentially expressed genes and a total of 784 gene sets related to CAFs infiltration were obtained (Fig. 1D and E). KEGG pathway analysis showed that the above gene sets were also significantly enriched into signal transduction and endocrine system related pathways (Fig. 1F).

### Construction and validation of CAFsbased risk model

**Fig. 2:**
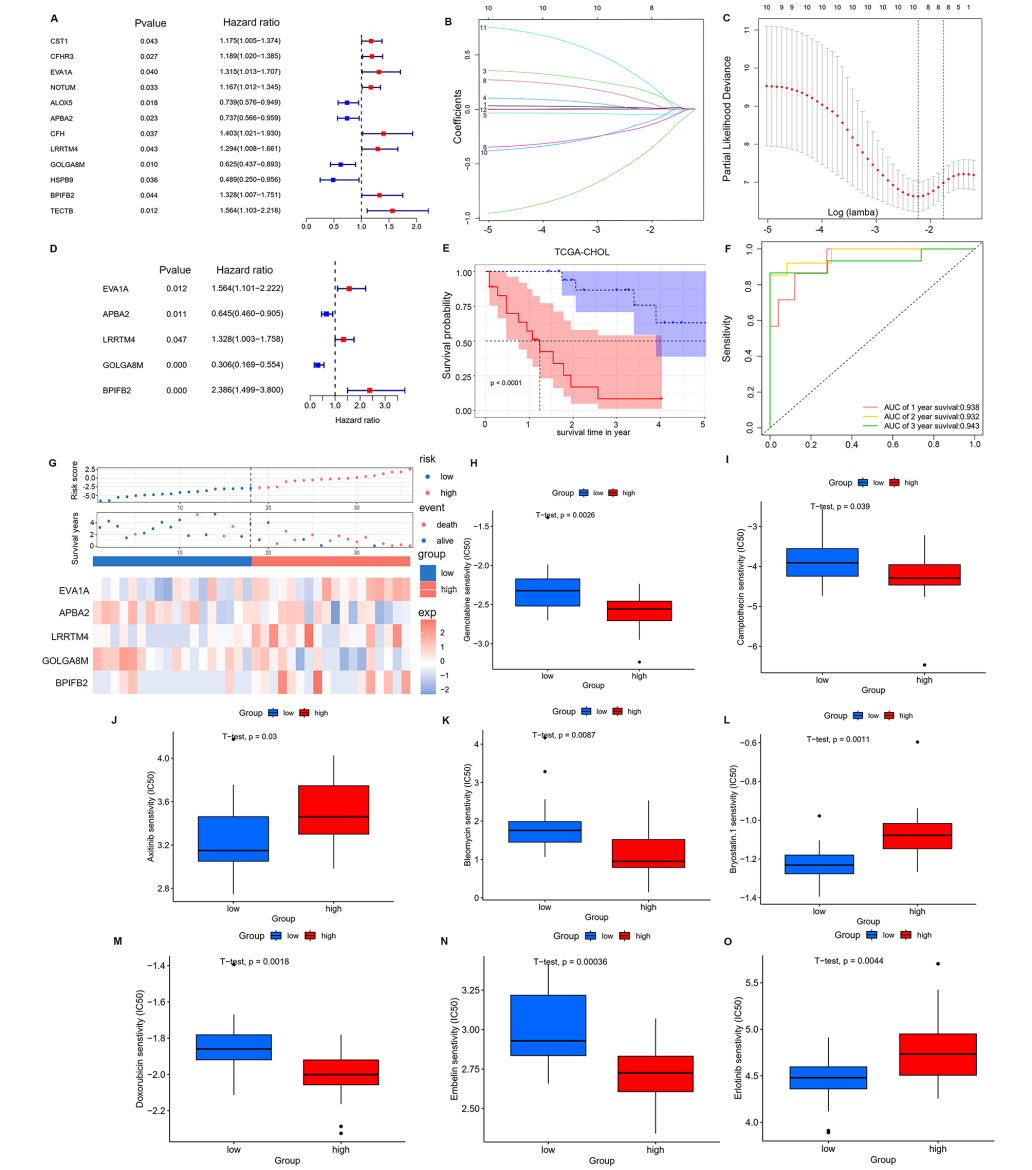
CAFs-related risk model identification and chemosensitivity analysis in the training and validation cohorts. (**A**) Twelve prognostic genes were screened from the 784 genes by univariate Cox analysis. (**B-C**) Coefficient distribution plots of log (lamda) sequences (**B**) and selection of optimal parameters (lambda) in the LASSO model (**C**). (**D**) Five prognostic genes were screened after multivariate Cox analysis. (**E**) Survival probability between patients with high and low CAFs scores. (**F**) ROC curves of CAFs scores in the training set. (**G**) Risk score distribution, survival status and genes expression patterns for patients in high- and low-CAFs scores groups. (**H-I**) Association between the CAFs scores and chemosensitivity in TCGA cohort. The box plots of the estimated IC50 for Gemcitabine, Camptothecin, Axitinib, Bleomycin, Bryostatin.1, Doxorubicin, Embelin, and Erlotinib were shown in the two groups

The TCGA-CHOL database with prognostic information on survival time and state served as the training set for constructing a CAFsbased risk model using genes obtained from the intersection process. Firstly, 12 CAFs-related genes significantly associated with survival were identified through univariate Cox proportional regression analysis (Fig. 2A). These genes underwent LASSO analysis for range reduction (Fig. 2B and C). Subsequently, nine genes were selected from the LASSO analysis, and after multivariate Cox regression analysis, only five genes remained (EVA1A, APBA2, LRRTM4, GOLGA8M, BPIFB2, *p* < 0.05) (Fig. 2D). The Coefficient β and gene expression levels of each gene obtained from multivariate Cox analysis were used to establish a CAFs-based risk model: CAFs score = (exp (EVA1A) * 0.447 - exp (APBA2) * -0.438 + exp (LRRTM4) *0.284 - exp (GOLGA8M) *1.183 + exp (BPIFB2) *0.870. Patients in the training set were stratified into high (*n* = 18) and low CAFs score group (*n* = 18) based on the median cutoff value. Notably, those with a high CAFs-score demonstrated a poorer prognosis (Fig. 2E and G). The predictive performance of the risk model was evaluated through a time-dependent ROC analysis, yielding AUC values of 0.938, 0.932, and 0.943 at 1, 3, and 5 years, respectively (Fig. 2F). Furthermore, patients with a higher CAFs-score exhibited increased sensitivity to Gemcitabine, Camptothecin, Bleomycin, Doxorubicin and Embelin (Fig. 2H, I, J, K, L, M, N and O).

**Fig. 3:**
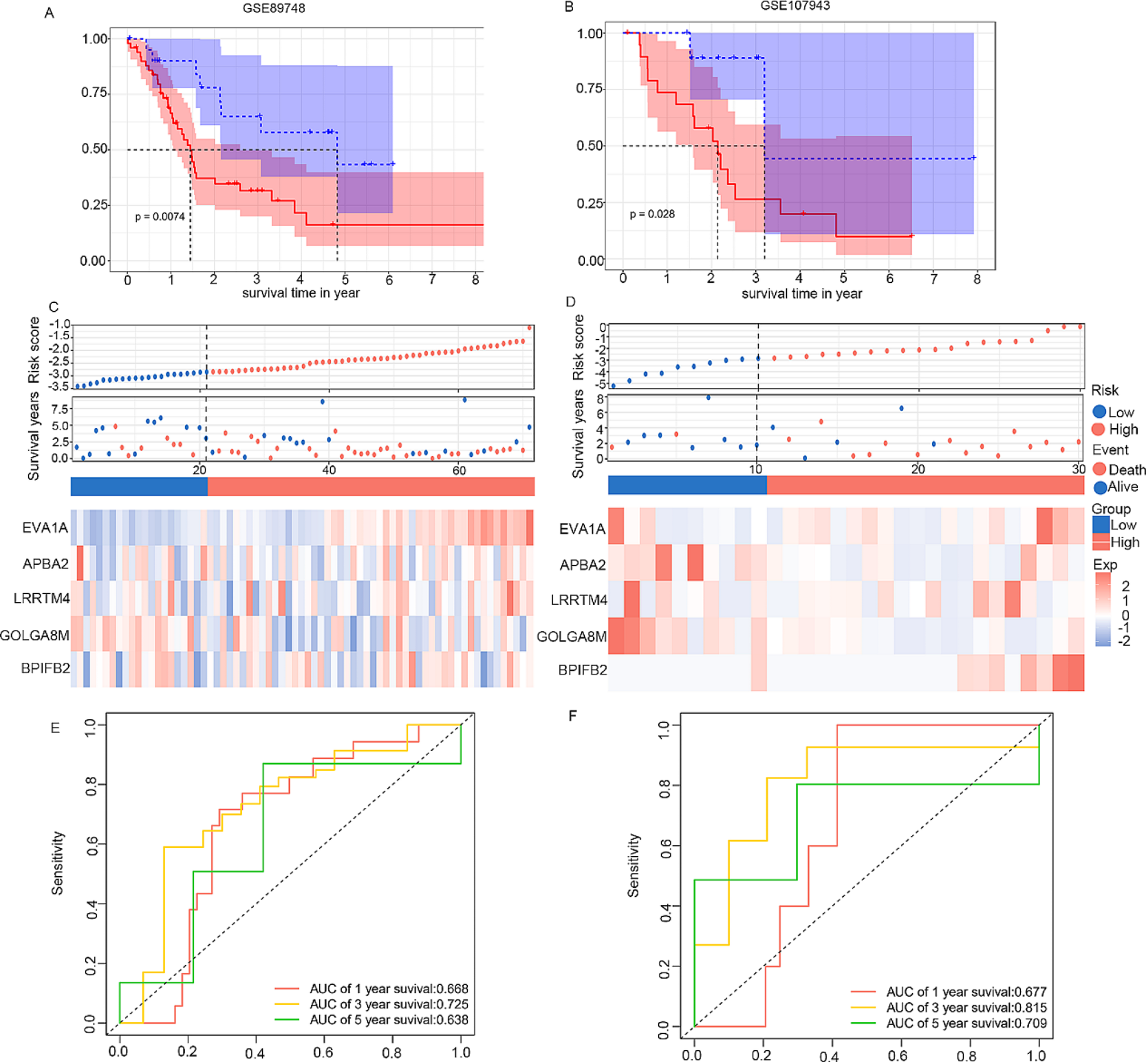
Validation of the risk signature for survival prediction in GSE89748 and GSE107943 sets. (**A-B**) Kaplan–Meier curve analysis of overall survival in two validation cohorts. (**C-D**) Risk score distribution, survival status and genes expression patterns in high- and low-CAFs scores groups. (**E-F**) Time-dependent ROC curves analysis

In the validation phase, GSE8974 and GSE107943 were utilized to confirm the predictive efficacy of the CAFs-based risk model. The risk score for each sample was computed using the aforementioned formula, with the cutoff value consistent with that of the training cohort. Kaplan–Meier analysis and risk plots consistently illustrated that patients with a high CAFs-score experienced a shorter survival time compared to their low-score counterparts in both GEO databases (Fig. 3A, B, C and D). Additionally, the ROC curve affirmed the reliable performance of the model across the two validated datasets (Fig. 3E and F).

### scRNA-seq data processing and cell type annotation

**Fig. 4:**
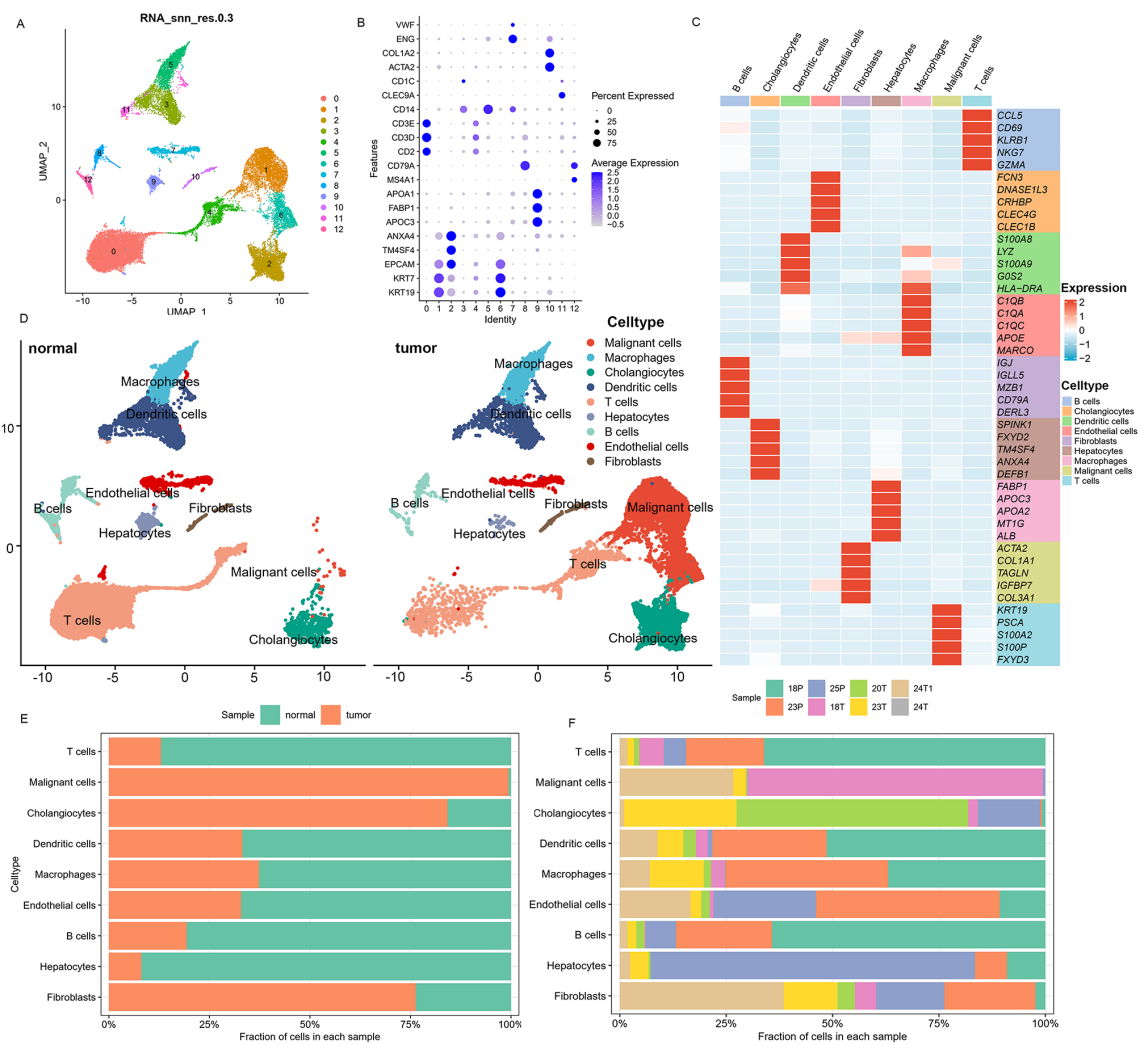
Single-cell Atlas of adjacent normal and tumor tissues. (**A**) UMAP plot for 13 distinct cell subclusters. (**B**) Dot plots depicting average expression of known markers in indicated cell clusters. (**C**) Heatmap showing the cell-type-specific top 5 DEGs (Wilcoxon test). (**D**) UMAP plots of cells from adjacent normal and tumor tissues of 5 ICC patients showing 9 clusters in each plot. (**E-F**) Proportion of 9 major cell types showing in bar plots in different tissues (**E**) and donors (**F**)

To unravel the intricate cellular composition of the tumor microenvironment and facilitate subsequent analyses, we annotated five tumor samples and three adjacent normal samples from five patients. Employing descending and unsupervised cell clustering, we identified cell subclusters based on their expression profiles. The Seurat package facilitated the reading of the raw dataset. Subsequently, an initial screening of genes and cells was conducted with the criteria that a gene had to be expressed in at least three cells, and each cell needed to measure between 200 and 6000 genes. Further quality control ensued, removing cells with mitochondrial genes exceeding 10% and ribosomal genes surpassing 3%, while also considering the expression of the MALAT1 steward gene. Next, we applied well-defined marker genes to characterize broad cell categories, leading to the identification of nine major cell subpopulations: Malignant cells, Hepatocytes, cholangiocytes, endothelial cells, dendritic cells, T cells, B cells, macrophages, and fibroblasts (Fig. 4A and B). The differentially expressed genes in each cell subgroup are shown in the Fig. 4C. Moreover, the infiltration proportion of these major cell types varied by tissue type and sample source, potentially indicative of distinctions in the stage of ICC progression (Fig. 4D, E and F).

### Characteristic analysis of cell subpopulations within CHOL

**Fig. 5:**
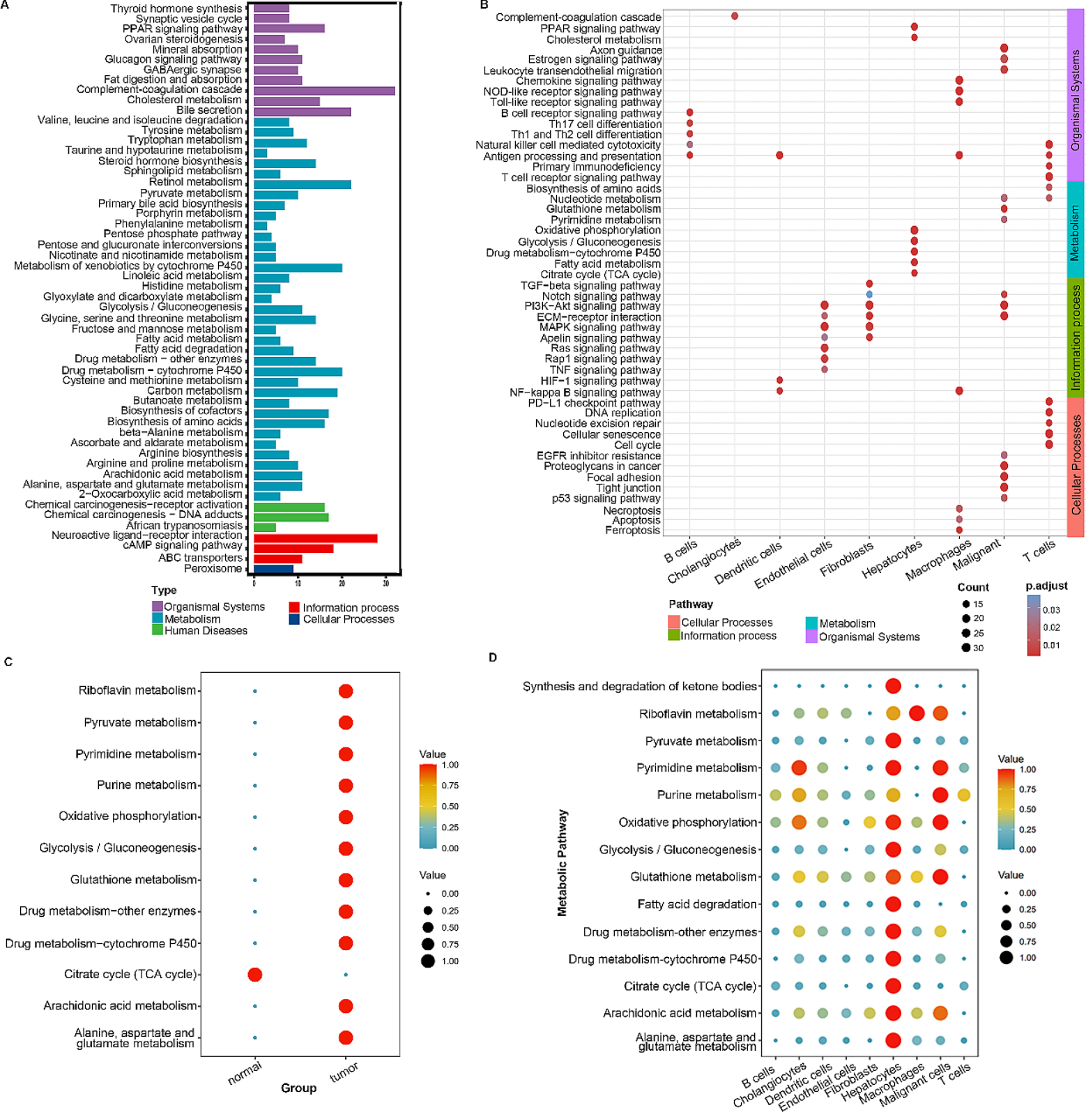
Pathway analysis in sample types and cellular subpopulations. (**A**) KEGG analysis of the Single cell global differentially expressed genes. (**B**) Functional annotation of nine cellular subpopulations. (**C**) Metabolic-related pathways comparison between tumor and adjacent normal tissues. (**D**) Dot plots show the specific metabolic pathways that were enriched in each cell subpopulation

To investigate pathway heterogeneity across distinct cell subpopulations, we conducted KEGG analysis utilizing signature genomes. According to the enrichment results, the pathways were broadly categorized into five groups, with a predominant emphasis on metabolism-related pathways (Fig. 5A). Notably, Hepatocytes, Malignant cells and T cells exhibited significant enrichment in metabolism-related pathways, including TCA cycle, Fatty acid metabolism, Glycolysis/Gluconeogenesis, Drug metabolism-cytochrome P450, Oxidative phosphorylation, suggesting a reprogramming of immunometabolism within the ICC TME. In addition, the environmental information processing related pathways, including ECM − receptor interaction, PI3K − Akt, Notch and TGF − beta signaling pathways, were mainly enriched in Endothelial cells, Fibroblasts and Malignant cells. This characteristic also reflects cell interaction that links Endothelial cells, Fibroblasts, and Malignant cells might remodel the ICC microenvironment. Moreover, cellular process related pathways, including Ferroptosis, Apoptosis and Necroptosis enriched in Macrophages, the p53 signaling pathway enriched in Malignant cells, and Cellular senescence enriched in T cells might involve in the processes of tumor cell proliferation and death (Fig. 5B). Taken together, these observations highlight the complex functional regulatory networks may impact the tumor microenvironment and contribute to tumor development through multiple mechanisms. Next, exploring metabolic differences between tumor and adjacent normal tissues involved scoring each metabolic pathway using the scMetabolism package. Strikingly, we observed an opposite trend, with the tumor subgroup exhibiting enrichment in the majority of metabolic pathways. This enrichment was particularly notable in pathways regulating Glycolysis/Gluconeogenesis, Oxidative phosphorylation, Citrate cycle (TCA cycle), and the metabolism of Pyruvate, Glutathione, and Drug metabolism related to cytochrome P450 (Fig. 5C). Upon further examination at the level of individual cell subpopulations, above significant differences may be related to Hepatocytes in the tumor samples (Fig. 5D).

### Analyzing differential intercellular communication between tumor and adjacent normal tissues

**Fig. 6:**
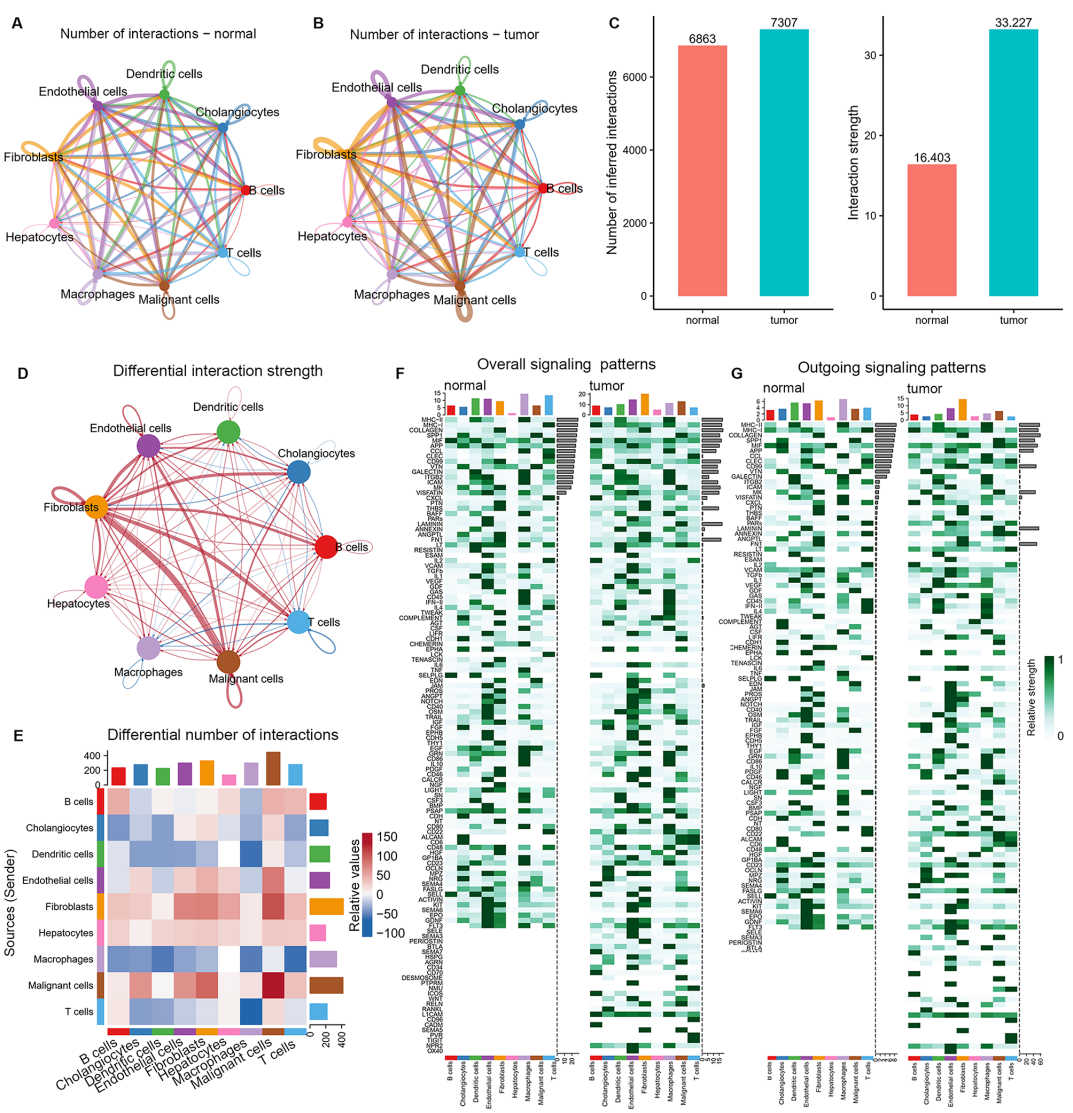
Comparison of cellular interactions between samples from tumor and adjacent normal tissues. (**A-B**) Cellular interaction number and strength. (**C**) Bar graph illustrating the total number (left) and weight (right) of ligand − receptor interactions between samples from tumor and adjacent normal tissues. (**D-E**) Communication quantity and intensity differences network. Red and blue colors represent upregulated and downregulated pathways, respectively, relative to normal tissues (**D**). (**F-G**) Heatmap showing possible afferent or efferent signaling pathways between cells

**Fig. 7:**
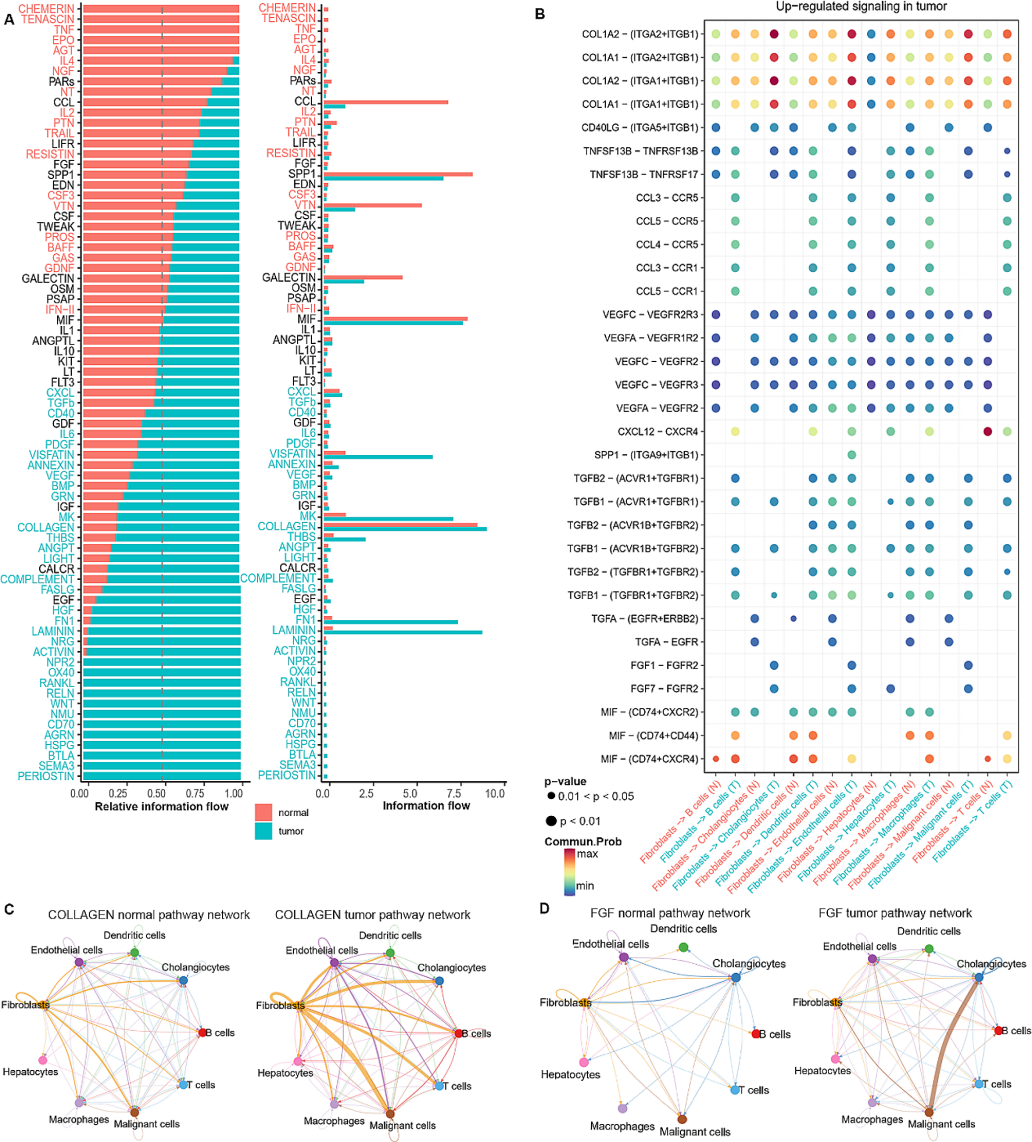
Comparison of cellular interactions between samples from tumor and adjacent normal tissues. (**A**) Comparative profiles of pathway signal intensities indicating conserved and specific signaling pathways in tumor and normal tissue samples. (**B**) Dot plots show the variation in the signaling action of fibroblasts relative to other cell types. (**C-D**) The COLLAGEN (**C**) and FGF (**D**) signaling pathway network in normal and tumor

We employed CellChat’s curated Secreted Signaling and ECM-Receptor databases to analyze cell interactions in single-cell datasets. Firstly, a communication network delineating alterations in signaling pathways between tumor and normal samples was constructed (Fig. 6A and B). Figure 6C illustrates variations in the number of communications across all cell populations between tumor and normal samples. In the tumor group, the number and strength of signaling pathways in fibroblasts to malignant cells, endothelial cells, hepatocytes, cholangiocytes, and T/B cells were significantly higher than those in the normal. However, Cellular communication from antigen-presenting cells, like macrophages and dendritic cells, to T cells was markedly reduced (Fig. 6D and E). Summarily, tumor samples demonstrated increased cellular interactions compared to normal counterparts, with a more pronounced impact on the overall signaling pattern. Additionally, the heatmaps also revealed fibroblasts contributed the most to overall information flow in both samples (Fig. 6F). Subsequently, we delved into the potential efferent and afferent signals within these nine cell types, specifically examining molecular pairs. Fibroblasts were the primary signal senders, whereas macrophages and malignant cells represented the main receptors in normal and tumor samples, respectively (Fig. 6G). The summary of communication probabilities in the information network facilitated a comparison of overall information flow differences between tumor and normal subgroups. Within normal samples, multiple pathways, such as those associated with CCL, TNF, and MIF, are engaged in intercellular signal transduction via the mediation of ligand receptor pairs. Conversely, in tumor samples, intercellular interactions were predominantly active in COLLAGEN, WNT, CXCL, TGFb, and EGF signaling pathways (Fig. 7A). Considering the pivotal role of fibroblasts in intercellular communication within ICC and their communication heterogeneity across tissues, we additionally assessed the frequency of ligand-receptor communication between fibroblasts and other cell types in both groups. The findings highlight the pivotal role of fibroblasts in orchestrating cell-cell communication within ICC, revealing diverse communication patterns across various tissues and cell types. Furthermore, ligand-receptor pair analysis found that fibroblasts preferentially sent signals to cholangiocytes, endothelial cells, and malignant cells by COL1A2-(ITGA2 + ITDB1) (L-R) in tumor samples, which influenced the tumor microbiome through intercellular information exchange and then mediated the immunosuppression and progression of ICC. Moreover, MIF-(CD74 + CD44) signaling between fibroblasts and cholangiocytes, dendritic cells and T cells was significantly enhanced in tumor tissue. Studies have shown MIF signaling pathway inhibits tumor apoptosis and promotes angiogenesis by activating downstream PI3K/Akt signaling pathway [24]. Specific to FGF signaling, it is mainly the ligand-receptor pair FGF1-FGFR2 that play a role in mediating the intercellular communication between fibroblasts and cholangiocytes, endothelial cells and malignant cells (Fig. 7B). Signal transduction between fibroblasts and other cells mediated by COLLAGEN pathway was notably amplified in tumor samples (Fig. 7C). A similar phenomenon occurs in the FGF signaling pathway (Fig. 7D). Additionally, several chemokine pathways, including CCL3-CCR1/5 and CXCL12-CXCR, showed significant augmentation in tumors. In summary, the complex intercellular signaling network in the tumor microenvironment is orchestrated by ligand-receptor pairs, suggesting potential targets for tumor therapy.

### Functional analysis of distinct fibroblast subpopulations

**Fig. 8:**
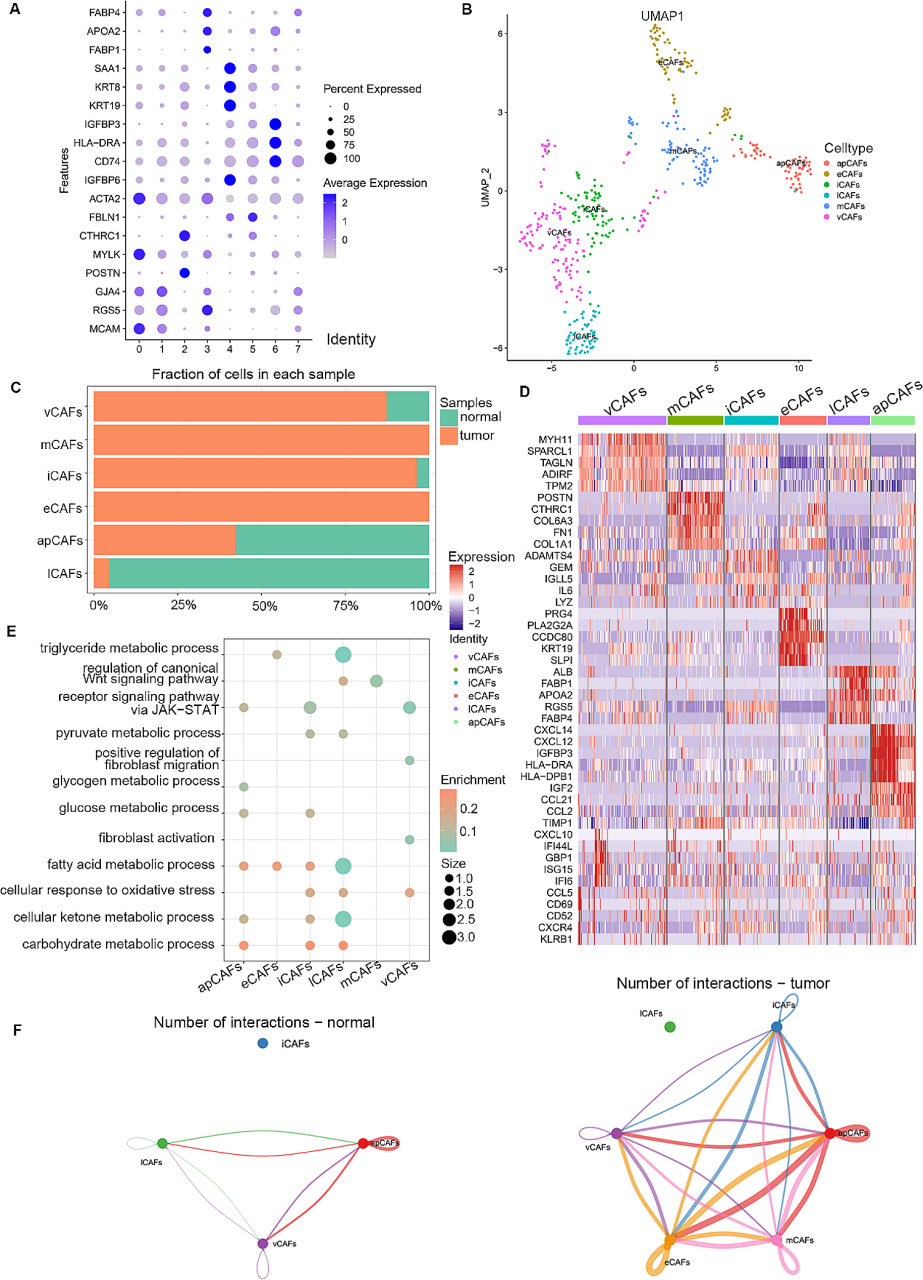
Characterization of fibroblasts subgroups in normal and tumor tissues. (**A**) Dot plot showing marker genes in each cluster. (**B**) UMAP plots of six different fibroblasts subpopulations. (**C**) Distribution of different fibroblasts subgroups from diverse sample origins. (**D**) Heatmap of the top 5 differentially expressed genes (DEGs) across six fibroblasts clusters. (**E**) Dot plots show the pathways that were enriched in each fibroblasts subpopulation. (**F**) Cellular interaction number and strength in normal and tumor groups

In the preceding analysis, it was evident that fibroblasts played a pivotal role in the comprehensive information exchange, with a notable surge in the interaction dynamics between fibroblasts and malignant cells observed in tumor tissues. For a detailed representation of their inherent features, these fibroblasts were intricately categorized into six subclusters utilizing the SNN algorithm in conjunction with t-SNE analysis (Fig. 8A and B). It is worthy to note that eCAFs is only detected in tumor samples. Other subgroups of CAFs other than eCAFs were distributed in tumors and adjacent normal tissues in each sample, but the degree of infiltration of each major cell type was different (Fig. 8C), which may reflect differences in the stage of ICC progression. The top 5 unique gene signatures of each fibroblast subgroups were delineated (Fig. 8D). Moreover, KEGG analysis of the CAFs subgroups showed that tumor-specific eCAFs were predominantly enriched in fatty acid and triglyceride metabolic process. CellChat analysis identified specifically activated WNT signaling pathways in tumor samples, which may be regulated by lCAFs and mCAFs (Fig. 8E). Compared with normal samples, the interactions among CAFs subtypes were more intensive and signal communication involving iCAFs was exclusively observed in tumor. In addition, more information was transmitted between apCAF, eCAFs and mCAFs in tumor samples (Fig. 8F).

### Transcription factor analysis of fibroblast infiltration associated genes

**Fig. 9:**
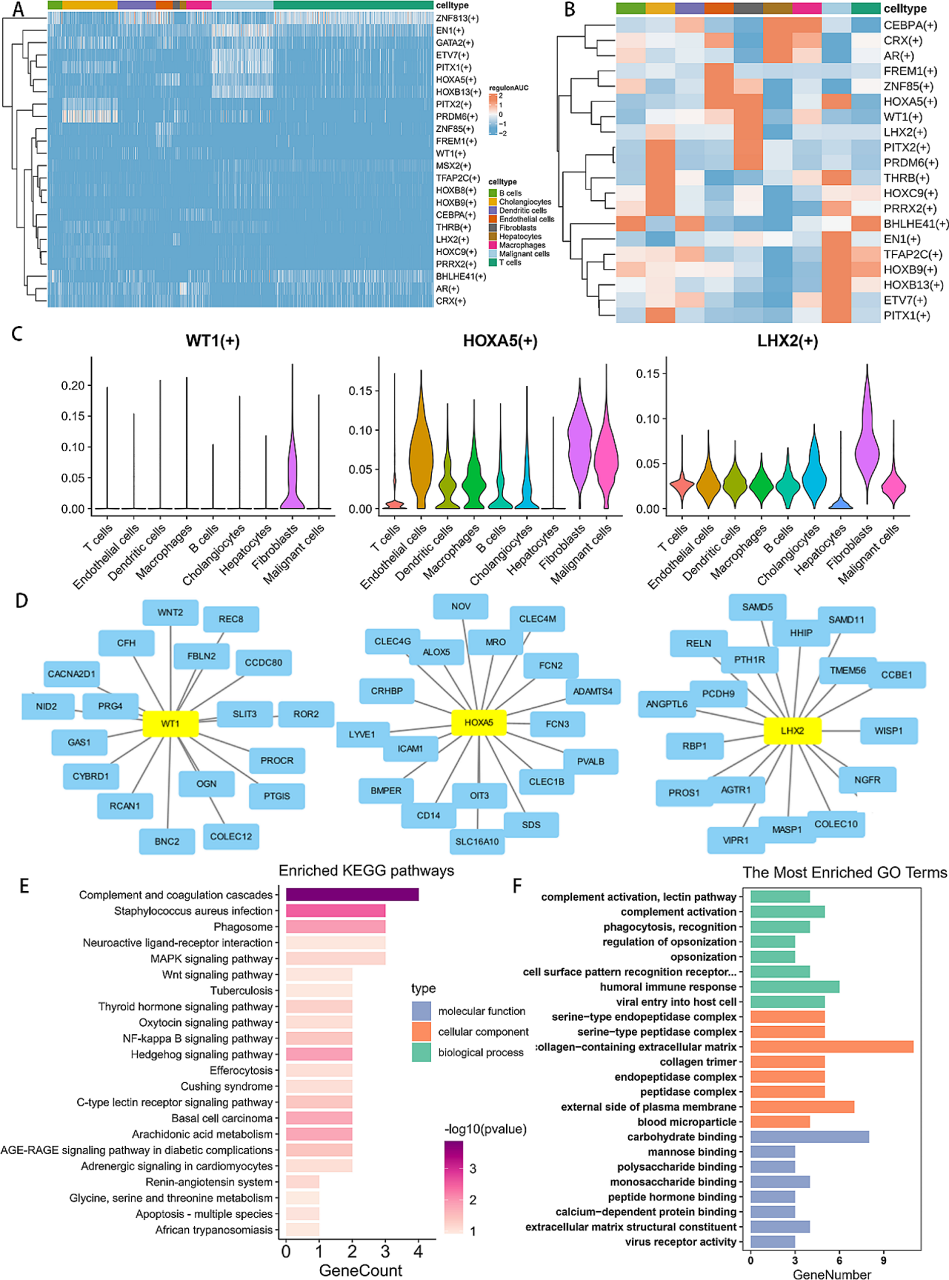
Transcription factor analysis of fibroblast infiltration associated genes. (**A**) Heatmap of the AUC scores of transcription factors (TFs) motifs in each cell subtype estimated per cell by pySCENIC. (**B**) Heatmap shows the specific transcription factors (TFs) in each cell subtypes. (**C**) Violin diagram showing the expression levels of transcription factors. (**D**) Fibroblasts-specific transcription factors and their targeted genes. (**E-F**) KEGG and GO analysis of the fibroblasts-specific transcription factors and their targeted genes

Modulating the tumor microenvironment encompasses an intricate interplay of transcription factors (TFs) engaging with each other and their effectors, ultimately influencing downstream genes. We further explore specific transcription factors that target the CAF infiltration-related genes in single-cell datasets. Heat maps were generated to illustrate variations in regulatory networks for each cell subset. Among all the cells, ZNF813, PRDM6 and EN1 exhibited the highest regulon activity (Fig. 9A). In addition, HOXA5, WT1 and LHX2 were identified as fibroblasts-specific motifs (Fig. 9B). Figure 9C show the spatial distribution characteristics of transcription factors. KEGG enrichment analysis of the targeted regulatory genes of HOXA5, WT1 and LHX2 showed that the above three specific TFs and their regulatory targets were enriched in some classical tumor-associated pathways, such as MAPK, WNT, NF-kappa B signaling (Fig. 9E). Furthermore, GO enrichment analysis highlighted the involvement of these genes in the formation of a collagen-containing extracellular matrix. (Fig. 9F).

## Discussion

Intrahepatic cholangiocarcinoma (ICC), characterized by a dense fibrotic microenvironment and high malignancy [25], is associated with chemotherapy resistance and a very poor prognosis [26]. Recent studies have elucidated the role of fibroblasts in fostering immunotherapy resistance by engaging with immune cells and components in the tumor microenvironment (TME) [27]. As expected, we found the strongest correlation between fibroblast infiltration and clinical features compared to other immune cells, with the proportion increasing as the TNM stage progresses. Moreover, we obtained the critical genes linked to CAFs infiltration through intersection of the TCGA DEGs and WGCNA module genes. Simultaneously, the risk model established by using the above CAF-related hub genes showed satisfied predictive performance. Subsequently, single-cell analysis revealed differences in cell composition and abundant cell interactions between tumor and normal samples, which led to remodeling of tumor microenvironment and tumor development.

Studies indicated that fibroblasts are the dominant heterogeneous cell type in TME and contribute to tumor development and chemotherapy resistance [28]. Likewise, we observed a correlation between fibroblast infiltration and various adverse outcome parameters, coupled with significantly enhanced signaling associated with fibroblasts. Through the application of Weighted Gene Co-expression Network Analysis (WGCNA), we pinpointed module genes intricately connected to the infiltration of Cancer-Associated Fibroblasts (CAFs). After intersecting with TCGA differential genes, we obtain the candidate hub gene set. Finally, five genes independently associated with prognosis, including EVA1A, APBA2, LRRTM4, GOLGA8M and BPIFB2, were screened by LASSO-COX regression and risk models were finally established. To the best of our knowledge, the five-gene marker stands as the first exploration into the overarching molecular prognostic attributes associated with fibroblast infiltration in intrahepatic cholangiocarcinoma. And this prognostic model exhibited favourable predictive performance in both training and two validation datasets. For patients with advanced disease, gemcitabine and cisplatin is acknowledged as the foremost and efficacious first-line regimen, while second-line (FOLFOX) and adjuvant (capecitabine) systemic chemotherapy are also employed [11]. In our study, we assessed the sensitivities of groups with high and low CAFs scores to chemotherapy. Patients with higher CAFs-scores demonstrated heightened sensitivity to Gemcitabine, Camptothecin, Bleomycin, Doxorubicin and Embelin (*p* < 0.05). However, due to methodological limitations, it is not possible to fully evaluate whether the CAF group is a good differentiator of therapeutic sensitivity to commonly used chemotherapy drugs. These existing observations provide insights into potential chemotherapy resistance mechanisms. The physical barrier created by excessive fibrosis surrounding the tumor impedes drug penetration, while blood vessels compressed by fibrous tissue make drug delivery more challenging [26]. Additionally, abnormal drug metabolism - cytochrome P450 pathways was noticed in hepatocytes, which could rapidly inactivate tumor drug and related to the above chemotherapy resistance [29].

Metabolic reprogramming is a recognized hallmark of cancer, enabling tumor cells to satisfy elevated energy requirements [30]. KEGG analysis of CAF infiltration-related genes obtained from WGCNA showed that Amino acid, Lipid and Carbohydrate metabolism were significantly enhanced. Deregulation of metabolic pathways were mainly associated with hepatocytes, including Glycolysis/Gluconeogenesis, Fatty acid metabolism and TCA cycle. The metabolism-related pathways that are abnormally activated in these tumors provide energy for tumor cell growth and metastasis [31]. In our study, FGF1/7-FGFR2 mediates the signal transmission from fibroblasts to malignant cells. Subsequently, the activated FGF pathway sets off a cascade of its downstream PI3K-AKT and Notch signaling pathways, which were highly enriched in malignant cells, regulate epithelial-to-mesenchymal transition (EMT) and invasion [32].

Analysis of the single-cell data showed that the cell subsets varied according to different groups and patient sources, which may be related to the heterogeneity and remodeling of the tumor microenvironment during the tumorigenesis. To further explore the influence of cell interaction on the above processes, we performed cellchat analysis. Notably, highlighting fibroblasts as principal contributors to the overall signal flow within ICC emphasizes their crucial impact on the cancerous microenvironment. This revelation prompted an in-depth exploration of the variances in ligand-receptor pairs responsible for mediating signal transmission between fibroblasts and other cells across diverse states. In tumors, there was a significant increase in the activation of COL1A2-(ITGA2 + ITDB1), a ligand-receptor pair related to COLLAGEN. Previous studies have linked fibroblasts as the main source of collagen, and excessive extracellular matrix, particularly collagen accumulation, is closely correlated with poor prognosis in various tumors. Therefore, the cell interaction mediated by COLLAGEN signal stimulates fibroblasts to secrete collagen, which accumulated in and around the tumor, thus promoting tumor growth, metastasis or drug resistance. Specific to FGF signaling, particularly FGF1-FGFR2, showed significant activation only in tumor samples. Furthermore, FGFR2 overexpression is linked to a poor diagnosis and treatment response [33], and its rearrangement or fusion is concentrated in intrahepatic cholangiocarcinoma [34, 35]. Previous studies have shown that key downstream signaling pathways altered by FGF-FGFR activation are the PI3K-AKT-mTOR pathway [36], which plays a central role in multiple oncogenic signaling pathways that drive tumorigenesis and progression [37]. Especially, enhanced PI3K/AKT pathway activation was more commonly observed in FGFR2 mutant cells [38], suggesting FGFR2 as a potential therapeutic target for cholangiocarcinoma. Preclinical studies with futibatinib, an irreversible FGFR1–4 inhibitor, corroborate this prospect [39]. In this study, the enhanced interaction between MIF and CD74 + CD44 in cholangiocarcinoma may inhibit tumor apoptosis and promote angiogenesis by activating the downstream PI3K/Akt signaling pathway [24]. With respect to the CXCR4-CXCL12 axis, with high CXCR4 expression in human cholangiocarcinoma tissues, was implicated in cholangiocarcinoma development and progression [40]. These findings underscore the importance of cell interaction and changes in downstream signaling pathways in exploring the mechanisms of tumorigenesis and identifying potential therapeutic targets.

Our objective was to elucidate resilient gene regulatory networks and identify pivotal transcription factors (TFs) involved in shaping fibroblasts-infiltrated microenvironments, achieved through SCENIC analysis of single-cell RNA-seq data. HOXA5, WT1, and LHX2 are fibroblast specific motifs that are drivers of tumor microenvironment remodeling, and their potential targets were highly correlated with collagen - containing extracellular matrix in KEGG analysis. HOXA5 is a transcription factor which could regulate cell differentiation, proliferation, and apoptosis as well as tumorigenesis [41]. Studies have shown that HOXA5 acts as a tumor suppressor to inhibits tumor growth and recurrence by regulating the PI3K/AKT/mTOR signaling pathway in hepatocellular carcinoma [42]. Furthermore, HOXA5 could also inhibit the proliferation of extrahepatic cholangiocarcinoma through activating the p53 pathway [43]. WT1 is a bidirectional transcription factor with dual functions of inhibiting tumor growth and activating oncogene transcription [44]. As a protective factor, LHX2 induces the expression of inhibitors targeting WNT and MAPK/ERK signaling pathways to stop tumor progression. However, it is noteworthy that LHX2 was found to be downregulated in liver tumors, a phenomenon that may accelerate anti-tumor progression [45].

Similar studies have been conducted in the past, for example, Zhang M, et al. [46], Wang T, et al. [47] have delved into the correlation between fibroblasts and intrahepatic cholangiocarcinoma based on single-cell sequencing data. In contrast, our study integrated the transcriptome and single-cell data, and the differentially expressed genes related to fibroblast infiltration were screened by WGCNA and TCGA difference analysis. A prognostic model was thereby constructed, and it was found that the model performed well in predicting patients’ survival outcome and drug sensitivity analysis. Subsequent analysis showed that the intercellular communication between fibroblasts and tumor cells was significantly enhanced in tumor, and then cascaded activation of downstream pathways such as PI3K-AKT and Notch. The role and function of each cell subpopulation in the tumor microenvironment are also explored. This discovery unveils new pathways for cancer drug exploration, including the inhibition of vital intercellular interactions and their signaling pathways, alongside the targeting of genes linked to fibroblast infiltration.

## Conclusion

In conclusion, our study highlights the crucial role of the desmoplastic matrix, particularly driven by fibroblasts - oncocytes interaction, in contributing to the therapy resistance and unfavorable prognosis observed in intrahepatic cholangiocarcinoma. Drugs targeting the interaction between fibroblasts and oncocytes, along with the highly enriched pathways between them, may help reverse the fibrotic microenvironment and inhibit tumor growth, metastasis, and drug resistance. However, because our study used bioinformatics and limited samples and survival data, the underlying mechanisms necessitate further validation through the inclusion of experimental investigations and larger cohorts.

## Data Availability

The datasets generated during the current study are available in the TCGA database (https://portal.gdc.cancer.gov/), GEO database (https://www.ncbi.nlm.nih.gov/geo/).

## Abbreviations

ICC: Intrahepatic cholangiocarcinoma
CAFs: Cancer associated fibroblasts
ScRNA-seq: Single-cell RNA sequencing
TFs: Transcription factors
EMT: Epithelial-mesenchymal transition
WGCNA: Weighted correlation network analysis
